# Investigation of the optimal design of orthodontic mini-implants based on the primary stability: A finite element analysis

**DOI:** 10.15171/joddd.2019.013

**Published:** 2019-08-14

**Authors:** Amir Hooman Sadr Haghighi, Vahid Pouyafar, Ali Navid, Mahsa Eskandarinezhad, Tannaz Abdollahzadeh Baghaei

**Affiliations:** ^1^Department of Orthodontics, Faculty of Dentistry, Tabriz University of Medical Sciences, Tabriz, Iran; ^2^Department of Mechanical Engineering, Tabriz University, Tabriz, Iran; ^3^Department of Endodontics, Faculty of Dentistry, Tabriz University of Medical Sciences, Tabriz, Iran

**Keywords:** Orthodontic anchorage procedures, orthodontic appliance design, bone screws

## Abstract

***Background.*** The design of an orthodontic mini-implant is a significant factor in determining its primary stability and its clinical success. The aim of this study was to measure the relative effect of mini-implant design factors on primary stability of orthodontic mini-implants.

***Methods.*** Thirty-two 3-dimensional assemblies of mini-implant models with their surrounding bone were generated using finite element analysis software. The maximum displacement of each mini-implant model was measured as they were loaded with a 2-N horizontal force. Employing Taguchi’s design of experiments as a statistical method, the contribution of each design factor to primary stability was calculated. As a result of the great effect of the upper diameter and length, to better detect the impact of the remaining design factors, another set of 25 models with a fixed amount of length and diameter was generated and evaluated.

***Results.*** The diameter and length showed a great impact on the primary stability in the first set of experiments (P<0.05). According to the second set of experiments, increased taper angle in the threaded and non-threaded area decreased the primary stability. There was also an optimum amount of 2.5 mm for threaded taper length beyond which the primary stability decreased.

***Conclusion.*** It is advisable to increase the diameter and length if primary stability is at risk. In the second place, a minimum amount of taper angle, both in the threaded and non-threaded area with an approximate proportion of 20% of threaded taper length to MI length, would be desirable for MIs with a moderate size.

## Introduction

Mini-implants (MIs) have been increasingly utilized in orthodontic treatment during the last decade; they eliminate the need for patient compliance and inconvenient extraoral appliances.^1^ Unfortunately, 13.4–20.1% of MIs have been reported to loosen and fail soon after placement.^2^ Accordingly, many suggestions have been made to enhance the MI survival rate. Sufficient primary stability is one of the determining factors.^3-5^ A better primary stability is attainable by altering the design of MI by increasing the length and diameter,^3,6,7^ increasing the intrabony length of MI,^8^ modifying the thread pitch,^9^ modifying the taper shape as in cylindrical, conical or combination designs,^9,10^ eliminating threads in the MI neck,^11^ modifying the thread shape,^12^ fluting,^13^ and altering the thread depth.^14^ On the other hand, design alteration without mechanical support should be interpreted with caution.^15^ In vitro mechanical experiments are also challenging because of the inaccurate parameter control and differences between the samples. In addition, the vast number of required experiments makes comparisons impossible.^16^

As a solution to this problem, finite element analysis (FEA) is a manageable and flexible technique, particularly suitable for demonstrating mechanical characteristics of biomaterials and human tissues which cannot be evaluated in vivo. It has also become quite well-known in the field of dentistry, especially in orthodontics.^15,17^ Additionally, the outcomes of FEA correlate well with experimental data.^18^

Various study designs have been combined with FEA to evaluate MI design. Some studies have evaluated one or two design factors such as MI pitch,^19^ presence of cervical threads,^11^ exposure length of the MI,^20^ taper,^21^ MI length, MI diameter^15,22-25^ and thread configuration.^26,27^ Some have evaluated several design factors such as taper, thread depth, thread shape and diameter at the same time but the quantified significance of each design factor was not calculated.^9,12^ In a more pragmatic approach, the relative significance of each design factor such as length, diameter and thread properties have been investigated simultaneously.^28,29^ The increased number of design factors evaluated in a single experiment improves the generalizability of the calculated relative significance values. These values are specific to that experiment and are not comparable to studies with different sets of design factors.

Taguchi’s design of experiments is a statistical method employed to calculate the effect of each design factor on the primary stability by measuring the displacement on a limited number of stimulated models.^29^ In the present study, Taguchi’s design of experiments and FEA were used to investigate the role of various design factors in determining the primary stability of MIs. The aim of this study was to investigate the ideal mini-implant design by determining the relative contribution of the main design factors of an orthodontic mini-implant to the primary stability, including diameter, length, taper and length of the threaded area, taper and length of the non-threaded area, pitch and thread depth/diameter.

## Methods

Models simulating the mini-implant and surrounding bone were created utilizing ABAQUS (Version 6.14, Dassault Systèmes Simulia Corp., Providence, RI, USA). The mini-implant was inserted in a bone block measuring 10×10×10 mm. The bone block consisted of an upper layer measuring 2 mm in thickness, representing cortical bone and the lower layer representing spongy bone. All the materials were supposed to be linear, solid, homogeneous, elastic and isotropic. Material properties of bone and implant were obtained from previous studies (Table 1).^30^

**Table 1:** Material properties

**Material**	**Poisson’s ratio**	Young’s modulus (MPa*)
**Mini-implant (titanium alloy)**	0.3	102000
**Cortical bone**	0.35	9000
**Cancellous bone**	0.3	700

*Megapascal

**Figure 1:**
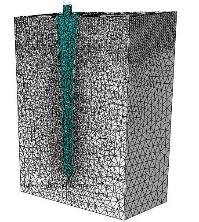
Meshing in one of the finite element models.

Mesh models were constructed by 4-node linear tetrahedral elements. Nodes on the surface of bone block were restricted to 3 degrees of freedom. The number of elements per model extended from 31,739 to 67,533 depending on the dimensions of the MI model. The interface between bone and mini-implant was defined as a “frictionless contact which allowed separation”, simulating non-osseointegrated state. A sample of meshing of the models is presented in Figure 1.

**Figure 2:**
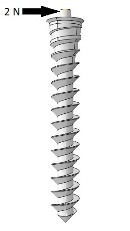
Application of a 2-N force at the head of the MI.

A horizontal force of 2 N was applied at the head of the mini-implant and the amount of maximum displacement was recorded for each model (Figure 2). The main effect of each design factor on the displacement was calculated at their corresponding levels.^31^

**Figure 3:**
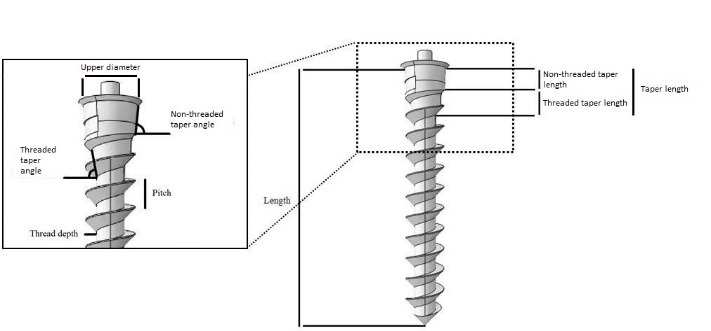
Mini-implant design factors.

The design factors assessed in this study were length, upper diameter, threaded taper length, threaded taper angle, non-threaded taper length, non-threaded taper angle, pitch and thread depth/diameter (Figure 3).

**Figure 4:**
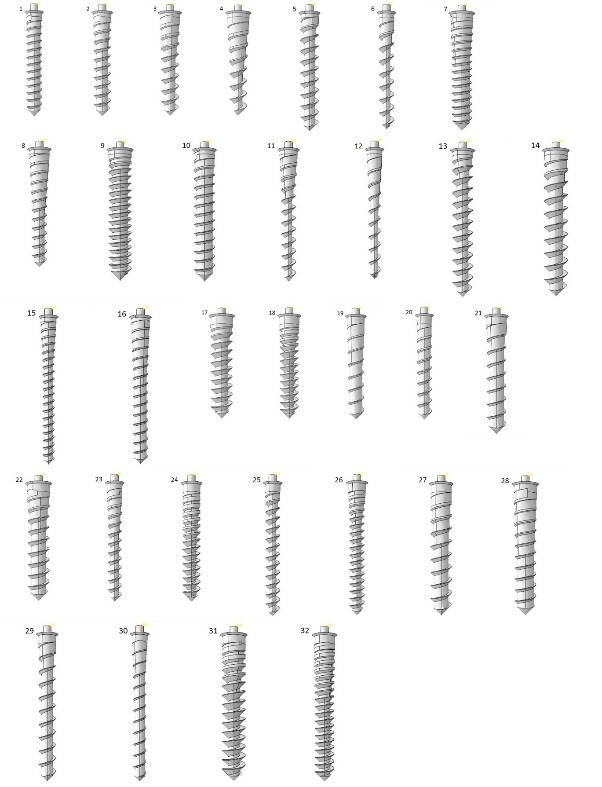
Thirty-two models of mini-implants for the Taguchi method, the first set of the models.

**Figure 5:**
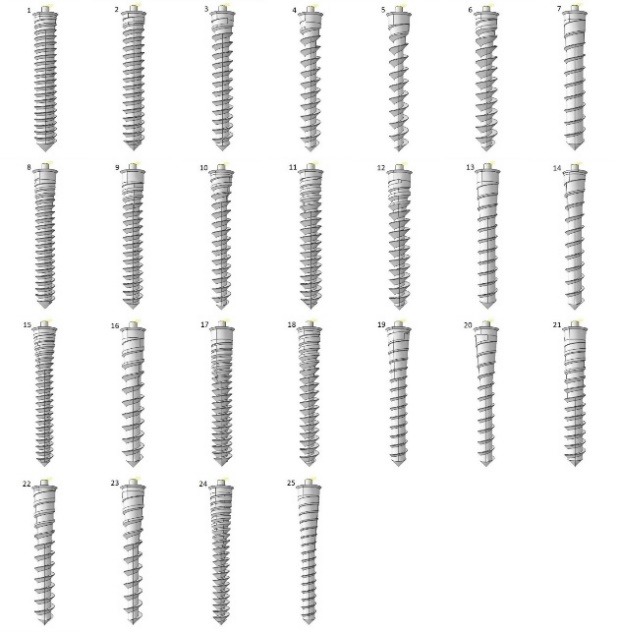
Twenty-five models of mini-implants for the Taguchi method, the second set of the models.

Two sets of FE models were generated and Taguchi’s design of experiments were carried out accordingly. The first one included 32 models with different combinations of length and upper diameter as well as threaded taper angle, threaded taper length, non-threaded taper angle, non-threaded taper length, pitch and thread depth/diameter (Table 2 and Figure 4). The maximum displacement of each model was measured and by means of Taguchi’s method, the main effect of each design factor on the displacement was calculated. The results showed a high significance for length and diameter while the effect of each remaining factor was <0.1%. In order to better detect the effect of the remaining design factors, a second set of models was generated. The length and diameter of the models were set to fixed amounts of 11 and 1.8 mm, respectively, and the number of assessed levels of the remaining design factors was increased (Figure 5 and Table 3). According to Taguchi’s method, the second set of models included 25 simulations. The same experiments were performed on the second set of models.

**Table 2:** Design factors and their according levels, first set of models

**Design factor**	**Level 1**	**Level 2**	**Level 3**	**Level 4**
**Length (mm)**	8	9	10	11
**Upper diameter (mm)**	1.2	1.4	1.6	1.8
**Threaded taper length (mm)**	1	2	3	4
**Threaded taper angle (˚)**	0	2	4	6
**Non-threaded taper length (mm)**	0.3	0.6	-	-
**Non-threaded taper angle (˚)**	0	2	4	6
**Pitch (mm)**	0.55	0.70	0.85	1.00
**Thread depth/Diameter**	0.1	0.15	0.20	0.25

**Table 3:** Design factors and their according levels, second set of models

**Design factor**	**Level 1**	**Level 2**	**Level 3**	**Level 4**	**Level 5**
**Threaded taper length (mm)**	**1.00**	**1.75**	**2.50**	**3.25**	**4**
**Threaded taper angle (˚)**	**0**	**2**	**4**	**6**	**8**
**Non-threaded taper length (mm)**	**0.1500**	**0.3125**	**0.4750**	**0.6375**	**0.8000**
**Non-threaded taper angle (˚)**	**0**	**2**	**4**	**6**	**8**
**Pitch (mm)**	**0.500**	**0.625**	**0.750**	**0.875**	**1.000**
**Thread depth/Diameter**	**0.10**	**0.14**	**0.18**	**0.22**	**0.26**

Analysis of variance was applied to determine the statistical significance of the difference between the effects of each design factor on the primary stability. The level of significance was set to α=0.05.

## Results

**Figure 6:**
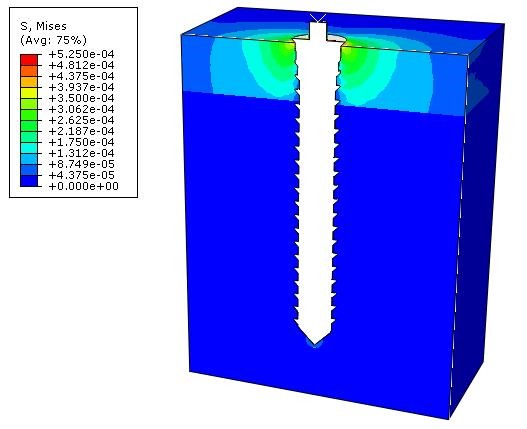
Stress distribution in the surrounding cortical bone.

The pattern of distribution of von mises stress in the surrounding cortical bone was the same for all the models (Figure 6).

Maximum displacements of the mini-implants are presented in Tables 4 and 5.

**Table 4:** Maximum displacement values of the mini-implant for each solved model, first set of experiments

**Model number**	**Length (mm)**	**Upper diameter (mm)**	**Threaded taper length (mm)**	**Threaded taper angle (˚)**	**Non-threaded taper length (mm)**	**Non-threaded taper angle (˚)**	**Pitch (mm)**	**Thread depth/diameter**	**Maximum displacement ×10**-^6^ **m**
1	8	1.2	1	0	0.3	0	0.55	0.10	121.2
2	8	1.4	2	2	0.3	2	0.70	0.15	112.4
3	8	1.6	3	4	0.3	4	0.85	0.20	105.2
4	8	1.8	4	6	0.3	6	1.00	0.25	99.0
5	9	1.4	1	0	0.3	2	0.85	0.20	103.6
6	9	1.2	2	2	0.3	0	1.00	0.25	113.3
7	9	1.8	3	4	0.3	6	0.55	0.10	88.5
8	9	1.6	4	6	0.3	4	0.70	0.15	97.2
9	10	1.8	1	2	0.3	4	0.55	0.15	81.7
10	10	1.6	2	0	0.3	6	0.70	0.10	87.7
11	10	1.4	3	6	0.3	0	0.85	0.25	97.6
12	10	1.2	4	4	0.3	2	1.00	0.20	106.0
13	11	1.6	1	2	0.3	6	0.85	0.25	84.4
14	11	1.8	2	0	0.3	4	1.00	0.20	77.0
15	11	1.2	3	6	0.3	2	0.55	0.15	99.1
16	11	1.4	4	4	0.3	0	0.70	0.10	87.9
17	8	1.8	1	6	0.6	0	0.70	0.20	95.6
18	8	1.6	2	4	0.6	2	0.55	0.25	103.3
19	8	1.4	3	2	0.6	4	1.00	0.10	112.7
20	8	1.2	4	0	0.6	6	0.85	0.15	123.5
21	9	1.6	1	6	0.6	2	1.00	0.10	95.1
22	9	1.8	2	4	0.6	0	0.85	0.15	87.7
23	9	1.2	3	2	0.6	6	0.70	0.20	113.9
24	9	1.4	4	0	0.6	4	0.55	0.25	101.9
25	10	1.2	1	4	0.6	4	0.70	0.25	105.4
26	10	1.4	2	6	0.6	6	0.55	0.20	97.2
27	10	1.6	3	0	0.6	0	1.00	0.15	86.9
28	10	1.8	4	2	0.6	2	0.85	0.10	81.3
29	11	1.4	1	4	0.6	6	1.00	0.15	91.0
30	11	1.2	2	6	0.6	4	0.85	0.10	98.6
31	11	1.8	3	0	0.6	2	0.70	0.25	75.8
32	11	1.6	4	2	0.6	0	0.55	0.20	81.6

m: meter, mm: millimeter

**Table 5:** Maximum displacement values of the mini-implant for each solved model, second set of experiments

**Model number**	**Threaded taper length (mm)**	**Threaded taper angle (˚)**		**Non-threaded taper length (mm)**	**Non-threaded taper angle (˚)**	**Pitch (mm)**	**Thread depth/diameter**	**Maximum displacement ×10-6 m**
**1**	1.00	0		0.1500	0	0.500	0.10	75.9
**2**	1.00	2		0.3125	2	0.625	0.14	76.8
**3**	1.00	4		0.4750	4	0.750	0.18	77.6
**4**	1.00	6		0.6375	6	0.875	0.22	78.5
**5**	1.00	8		0.8000	8	1.000	0.26	79.3
**6**	1.75	0		0.3125	4	0.875	0.26	77.0
**7**	1.75	2		0.4750	6	1.000	0.10	77.1
**8**	1.75	4		0.6375	8	0.500	0.14	77.6
**9**	1.75	6		0.8000	0	0.625	0.18	76.3
**10**	1.75	8		0.1500	2	0.750	0.22	79.2
**11**	2.50	0		0.4750	8	0.625	0.22	76.7
**12**	2.50	2		0.6375	0	0.750	0.26	76.0
**13**	2.50	4		0.8000	2	0.875	0.10	76.5
**14**	2.50	6		0.1500	4	1.000	0.14	78.4
**15**	2.50	8		0.3125	6	0.500	0.18	78.4
**16**	3.25	0		0.6375	2	1.000	0.18	76.2
**17**	3.25	2		0.8000	4	0.500	0.22	76.2
**18**	3.25	4		0.1500	6	0.625	0.26	77.6
**19**	3.25	6		0.3125	8	0.750	0.10	78.4
**20**	3.25	8		0.4750	0	0.875	0.14	78.0
**21**	4.00	0		0.8000	6	0.750	0.14	76.7
**22**	4.00	2		0.1500	8	0.875	0.18	77.5
**23**	4.00	4		0.3125	0	1.000	0.22	77.7
**24**	4.00	6		0.4750	2	0.500	0.26	77.3
**25**	4.00	8		0.6375	4	0.625	0.10	78.5

m: meter, mm: millimeter

**Figure 7:**
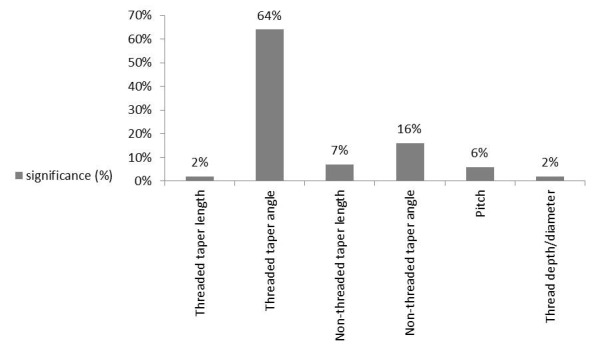
Significance (%) of each design factor for the given length and diameter of 11 and 1.8 mm, respectively (second set of experiments).

According to the first set of experiments, upper diameter (an effect of 53% resulting from diameter change from 1.2 to 1.8 mm) and mini-implant length (an effect of 45% resulting from length change from 8 to 11 mm) were the main design factors determining maximum displacement (P=0.000 for both). The percentage of contribution to primary stability for the remaining factors was <0.1%, which was statistically insignificant (P>0.05). The effect of each design factor in the second set of experiments was statistically significant (P<0.05). The percentage of the effect of each design factor is presented in Figure 7.

**Figure 8:**
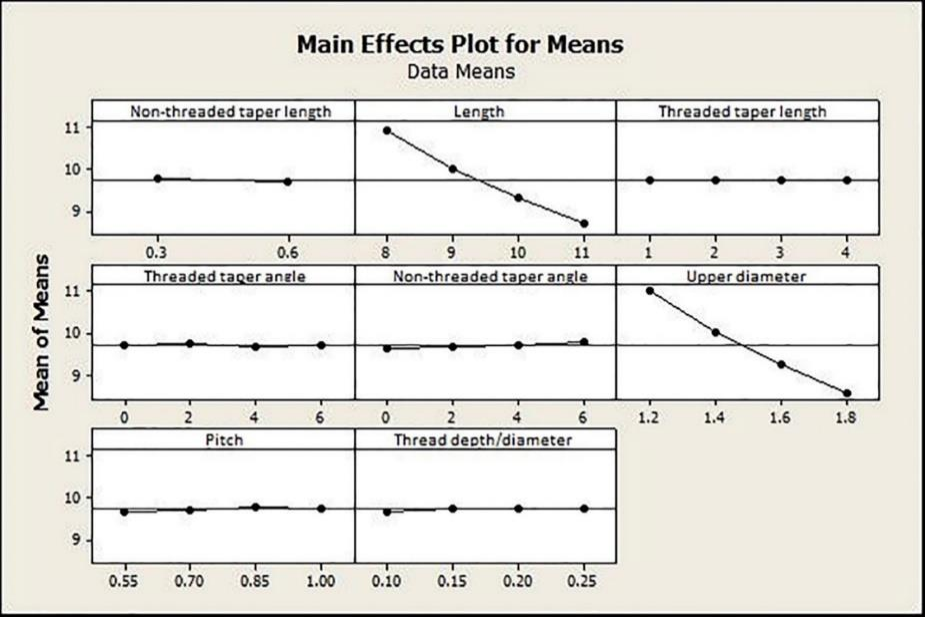
Main effects plot for means of each design factor, the first set of experiments.

**Figure 9:**
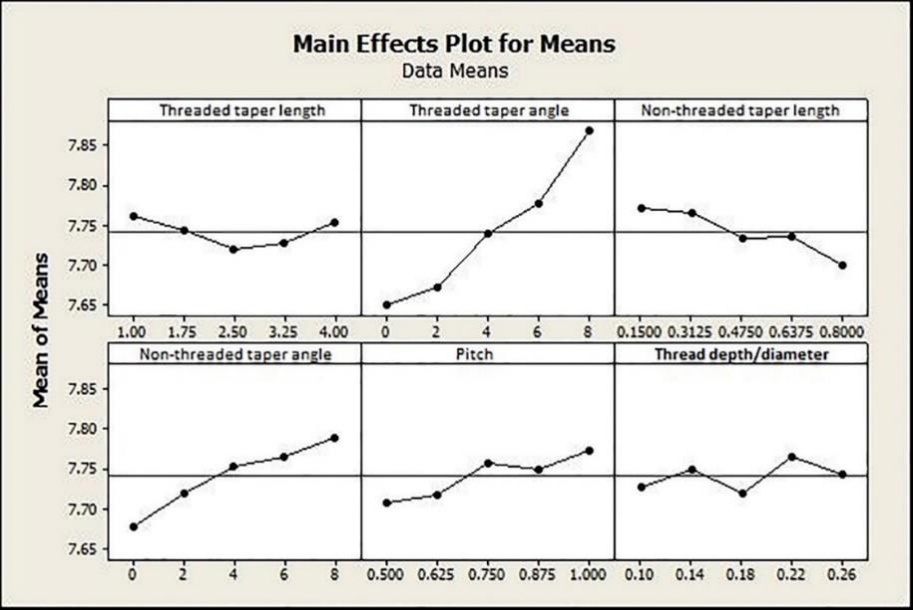
Main effects plot for means of each design factor, the second set of experiments.

The main effects plot for maximum displacement also showed the significant impact of upper diameter and length on MI displacement (Figure 8). According to Figure 9, maximum displacement increased as threaded taper angle and non-threaded taper angle increased and decreased as non-threaded taper length increased. The maximum displacement was the highest as pitch value reached 1 mm. Threaded taper length showed an optimum value of 2.5 mm. Thread depth/diameter did not show a definitive pattern.

## Discussion

Many parameters have been applied as an indicator of clinical success in mechanical experiments. The relationship of frequently used factors such as stress, strain and insertion torque with clinical success is under question. In order to achieve more practical results, the primary stability which determines the clinical success was considered as the main outcome of this study.^5^

A non-threaded area as a new design feature was also tested among the other design factors in the MI design, which is believed to contribute to primary stability. Calculation of the relative influence of each design factor would have required measurement of maximum displacement in thousands of different MI design combinations. By employing Taguchi’s design of experiments, the number of simulations decreased to 32 for the first set of experiments and 22 for the second set.

### Diameter and Length

The significant effect of diameter and length, respectively, on primary stability was confirmed in this study. The dominant role of diameter relative to length in decreasing stress and displacement has also been noted.^15,25,29,32^ These findings are also consistent with higher success rates reported for longer and larger mini-implants.^16^

Some studies have shown no relationship between length and success rate; this contradiction may have been a result of the confounding variables,^33,34^ insufficient sample size^35^ and loss of data.^36^ The results on the positive effects of length and diameter were consistent with some other in vivo investigations based on the survival rate.^37,38^

### Taper Length and Angle in the Threaded and Non-threaded Areas

Tapered MIs are intended to gain primary stability by generating a compressive force in the cortical bone. In this study, an increase in threaded taper angle and non-threaded taper angle resulted in a decrease in primary stability. This is attributable to the simultaneous decrease in the diameter and therefore bone‒mini-implant contact area, which occurs rapidly in higher taper angles. These findings imply that when comparing MIs with the same upper diameter, the one with less taper angle, either in the threaded or the non-threaded area, is more favorable.

Threaded taper length exhibited an optimum amount of 2.5 mm for an 11×1.8-mm mini-implant. While tapering improved the primary stability to an upper threshold, higher length of taper also decreased the diameter as it was for the threaded and non-threaded taper angle. The non-threaded taper length also affected the primary stability positively but the optimum amount was not achieved as the maximum amount tested was only 0.8 mm. Higher lengths of non-threaded area were not tested because it might have overridden the benefits of threaded design. Yoo et al^10^ reported higher primary stability for the tapered MIs, although this superiority was not manifested in the clinical success rates. In that study, the maximum diameter of the tapered MI was more than the cylindrical one. This makes the upper diameter a confounding factor and explains the different results.

Involvement of a non-threaded area of 0.8 mm would be a favorable modification in the MI design. Even if the MI design does not include a non-threaded area in the intrabony part, insertion of a mini-implant further off would contribute to the primary stability by increasing the intrabony length considering that the non-threaded area itself improves the primary stability, too. This finding was in agreement with another finite element study.^11^

### Pitch and Thread Depth/Diameter

The pattern of changes in pitch and thread depth/diameter did not show a definite pattern. These two factors were also the least effective ones (6% and 2%, respectively). Although there seems to be an inverse relationship between pitch and primary stability, there is controversy over the effect of pitch. Although no studies have assessed the survival rate or primary stability of MIs with different levels of pitch, some have compared the stress levels. Motoyoshi et al^19^ reported lower stress with decreased values of pitch, while another investigation showed different results;^39^ and one of them reported no relationship between pitch and stress levels in the cortical bone area.^12^

### Limitations

The boundary conditions in this study were defined as “frictionless contact with allowed separation” as the bone is not bonded or fully osseointegrated to the MI under clinical conditions. There is controversy over the choice of the contact area: frictionless or frictional. Different studies have employed different conditions and friction constants. Disagreement between studies still remains.

Another assumption of the present study was that all the materials were homogeneous, linear and isotropic while bone material is neither homogeneous nor isotropic.^24^ These assumptions made the assessment easier while sacrificing the validity of absolute values of mini-implant displacement in clinical conditions. The numerical results of FEA also greatly depend on mesh design, number of elements, interface area and many other technical factors. Accordingly, data derived from the present study was not compared to the results of other investigations and the pattern of changes in maximum displacements was taken into account instead of the definite amount of displacement.

### Recommendations

Further investigations by combining Taguchi method and FEA to analyze more design factors at the same time would help to precisely determine the ideal orthodontic MI design. Simultaneous in vitro and in vivo experiments to evaluate the mechanically approved designs would also be helpful.

## Conclusions

1. The contribution of MI diameter and length to the primary stability, relative to the other design factors, is substantial (53% and 45%, respectively). It is advisable to increase the diameter and length first if the primary stability is at risk.
2. A minimum amount of taper angle, with the optimum threaded taper length of 2.5 mm is desirable for an 11×1.8-mm MI (P<0.05). Consequently; an approximate proportion of 20% of threaded taper length to MI length would be desirable for similar sized MIs.
3. 0.8 mm of non-threaded area in combination with the threaded part increases the primary stability (P<0.05) and therefore, insertion of the MI beyond the uppermost thread is of benefit.
4. Mini-implants with the same upper diameter and less tapering are more stable than the highly-tapered ones due to the rapid decrease in the bone/mini-implant contact area in the tapered ones (P<0.05).
5. Thread pitch and thread depth have trivial effects on MI stability (P<0.05).

## Authors’ Contributions

AHSH and ME conceived and designed the analysis. VP contributed to data or analysis tools. AN performed the analysis. TAB prepared the paper and collected the data.

## Acknowledgments

None.

## Funding

Not applicable.

## Competing Interests

The authors declare no competing interests with regards to the authorship and/or publication of this article.

## Ethics approval

Not applicable.
